# Application of Proteomics to Inflammatory Bowel Disease Research: Current Status and Future Perspectives

**DOI:** 10.1155/2019/1426954

**Published:** 2019-01-15

**Authors:** Arash Assadsangabi, Caroline A. Evans, Bernard M. Corfe, Alan Lobo

**Affiliations:** ^1^Gastroenterology Unit, Salford Royal Hospital, Salford, UK; ^2^Molecular Gastroenterology Research Group, Academic Unit of Surgical Oncology, Department of Oncology and Insigneo Institute, University of Sheffield, Sheffield, UK; ^3^Department of Chemical and Biological Engineering, University of Sheffield, Sheffield, UK

## Abstract

Inflammatory bowel disease (IBD) is a chronic relapsing/remitting inflammatory illness of the gastrointestinal tract of unknown aetiology. Despite recent advances in decoding the pathophysiology of IBD, many questions regarding disease pathogenesis remain. Genome-wide association studies (GWAS) and knockout mouse models have significantly advanced our understanding of genetic susceptibility loci and inflammatory pathways involved in IBD pathogenesis. Despite their important contribution to a better delineation of the disease process in IBD, these genetic findings have had little clinical impact to date. This is because the presence of a given gene mutation does not automatically correspond to changes in its expression or final metabolic or structural effect(s). Furthermore, the existence of these gene susceptibility loci in the normal population suggests other driving prerequisites for the disease manifestation. Proteins can be considered the main functional units as almost all intracellular physiological functions as well as intercellular interactions are dependent on them. Proteomics provides methods for the large-scale study of the proteins encoded by the genome of an organism or a cell, to directly investigate the proteins and pathways involved. Understanding the proteome composition and alterations yields insights into IBD pathogenesis as well as identifying potential biomarkers of disease activity, mucosal healing, and cancer progression. This review describes the state of the art in the field with respect to the study of IBD and the potential for translation from biomarker discovery to clinical application.

## 1. Introduction

IBD is an idiopathic chronic condition of unknown aetiology with an inflammatory gut response to unidentified triggers. It broadly encompasses two major disease categories, namely, ulcerative colitis and Crohn's disease, with a relapsing and remitting course. In ulcerative colitis (UC), the mucosal inflammation affects the rectum with variable proximal colonic involvement but always in a continuous fashion. Although it only affects the colon, total colitis can be associated with a “backwash” ileitis. Crohn's disease (CD) is characterised by discontinuous areas of transmural inflammation. Although it can involve any part of the gastrointestinal tract, the terminal ileum and proximal colon are more frequently affected.

IBD is most common in Northern Europe and North America with a prevalence of approximately 400 per 100,000 in the UK [1, 2]. Low incidence areas include southern Europe, Asia, and most developing countries, although the rate of the disease is on the rise on these regions [2]. There has been an increase in the number of Scottish children diagnosed with IBD since the mid-1990s [3]. Men and women are affected similarly. There is a bimodal age of presentation with an initial peak in the second and third decades of life followed by another peak in the sixth decade [4].

Although the exact pathogenesis of IBD is still unknown, disturbance in the normal homeostasis between the intestinal barrier cells (including epithelial, mesenchymal, and immune cells) and commensal intestinal microbiota in a genetically susceptible host plays a central role in its development [5]. A large list of environmental factors, termed the exposome, have been proposed to have influence in the pathogenesis of IBD such as smoking [6], diet [7, 8], medications [9], exercise [10, 11], air pollution [12], breast feeding [13], excessive sanitation during infancy [14], and psychological stress [15]. The evidence for environmental triggers in IBD has recently been reviewed in detail with focus on the complex interactions between the exposome, genome, immune system, and microbiome, concluding that the gut microbiome is central to the pathogenesis of IBD [16].

Dysbiosis plays an important part in the pathogenesis of IBD. There are about 1100 prevalent bacterial species in the normal human gut microbiome with each individual harbouring at least 160 such species [17]. A significant reduction in butyrate producing-bacteria and increase in microorganisms with mucin degradation capacity have been observed in the gut microbiome of patients with IBD [18–20]. There is significant interest in the gut microbiome, with recent findings suggesting that mucosa-associated microbiota changes in Crohn's disease are more marked than faecal changes [21]. Whether dysbiosis is a primary or secondary event in IBD progression remains unclear.

GWAS and immunological studies have confirmed the important role of both innate and adaptive immunity in the pathophysiology of IBD [22, 23]. CD is long known to be mediated by Th1-mediated immune response as opposed to UC, which triggers a Th2-mediated response [24, 25]. Th17-mediated proinflammatory response via IL-23/IL-17 pathway as well as ineffective anti-inflammatory Treg-mediated response is contributory in IBD pathogenesis [26, 27]. On the other hand, the role of innate mucosal immunity such as epithelial barrier integrity, microbial sensing, autophagy, and unfolded protein response is becoming more evident in regulating an appropriate inflammatory response in IBD [28, 29]. Altered patterns of cytokine production from the innate and adaptive immune cells are in turn responsible for the continuation of intestinal inflammation and associated symptoms, as well as extra-intestinal manifestations of IBD [30].

There is considerable interest and benefit to the use of biomarkers for diagnosis and management of IBD. A biomarker is defined by the National Institutes of Health Biomarkers Definitions Working Group as “a characteristic that is objectively measured and evaluated as an indicator of normal biological processes, pathogenic processes, or pharmacologic responses to a therapeutic intervention” [31]. For IBD, biomarkers are of value in diagnostic and prognostic purposes with the potential to identify treatment strategies for disease management. Biomarkers to assess intensity of inflammation are also of value, as are those indicating disease course and associated complications [32]. Furthermore, risk stratification of subjects with a history of IBD into those likely to relapse and those who will remain quiescent remains a significant challenge. Risk and benefit assessment in IBD treatment is also a major issue especially as the disease behavior is unpredictably variable amongst patients; therefore, a reliable predictive biomarker would be very beneficial in stratifying aggressive immunosuppressive treatments to patients at high risk of severe relapses, while avoiding potential harmful drug exposure to those with more indolent disease course [32–34].

Protein molecular biomarkers or panels of biomarkers to form “biomarker signatures” are particularly popular for noninvasive testing. Proteomics enables biomarker discovery by providing methods for identification and quantification of proteins and thus comparative profiling of complex biological samples.

## 2. Proteomics

The term “proteomics” was coined in 1995 from the combination of genomics and proteins [35]. The proteome is the entire set of proteins that can be expressed by a genome in a cell. Proteomics encompasses studies for the identification of all proteins as well as their function and structure.

A general workflow applied in nearly all proteomic studies included in this paper involves sample selection, protein extraction, protein or peptide preparation, protein/peptide separation, protein/peptide identification and quantification, and statistical treatment of the quantitative data obtained and the interpretation of the results using bioinformatics [36, 37]. To date, there is no one method for proteomic analysis. In order to reduce the complexity of proteomic investigation, cellular compartmentalisation has been proposed [38]; these smaller cellular compartments are sometimes referred to as “secretome” (investigation of secreted proteins in bodily fluids or cell culture supernatants), “cell surface proteome,” “phosphoproteome,” and “interactome” (investigation of the set of protein-protein interactions taking place in a cell).

Briefly, identification of proteins in proteomics occurs through the analysis of (i) intact proteins or (ii) protein fragments termed “top-down” and “bottom-up” approaches, respectively. Top-down proteomic approaches are particularly suited to analysis of different protein products encoded by a single gene protein. These products are termed proteoforms, a single term per protein to encompass diversity [39] that can result from genetic variation, alternatively spliced RNA transcripts, and posttranslational modifications [40]. Generating an inventory of human proteoforms and multiproteoform complexes poses significant technical challenges as outlined by Naryzhny [39]. Bottom-up proteomics achieves protein identification by analysis of peptide fragments generated by proteolytic digestion of intact proteins and has been widely employed. Proteomics offers a versatile toolkit of analytical techniques. Top-down and bottom-up proteomic analyses yield complementary information. There are two core technologies employed in proteomic analysis: protein separation and analysis using mass spectrometry for protein identification, characterisation, and quantification.  Table 1 provides information on different proteomic techniques, with examples of key applications to IBD research and original papers describing their implementation and reviews of their use for clinical proteomics. For more information, there are review articles that provide an overview of commonly used proteomic techniques and workflows [41, 42].

## 3. Proteomic Techniques

The implementation of proteomic techniques has revolutionised biomarker discovery. For clinical proteomics, the commonly used quantitative proteomic methodologies are “gel-based” polyacrylamide gel electrophoresis (PAGE) and two-dimensional difference gel electrophoresis (2DE) and “gel-free” isotope-tagging/labelling technologies, including iTRAQ (isobaric tags for relative and absolute quantification), SILAC (stable isotope labelling with amino acids in cell culture), and so-called label-free quantification [43]. In general, gel-free methods can address many of the shortcomings of gel-based approaches, which is tedious and inefficient in resolving proteins that are low abundant, insoluble, or large (200 kDa) [44]. Protein identification from 2DE gels requires excision of individual protein spots of interest and processing for identification by MS, often based on peptide mass fingerprinting (measurement of peptide mass (mass/charge)) or MSMS analysis whereby peptides are fragmented to provide amino acid sequence and or posttranslational modification (PTM) information. Proteins are processed by proteolytic digestion to generate peptides; information from peptides is integrated to the protein level. Gel-free separation of proteins is performed at the peptide level, for technical reasons since peptide separation is more amenable to high throughput in the liquid phase than for proteins. High-performance liquid chromatography (HPLC) techniques are employed, typically reversed-phase HPLC. For complex samples, typically peptides, fractionation employs an additional HPLC step prior to reversed-phase HPLC, termed 2D-HPLC, using a different, orthogonal separation chemistry. In this case, an HPLC or UPLC method is selected to exploit different physico-chemical properties of peptides, e.g., charge or hydrophobicity, to achieve greater sample fractionation than can be achieved by reversed-phase HPLC alone, to boost the number of peptides and thus proteins identified in a sample. Such additional fractionation is typically performed off-line prior to LC-MS or MS/MS analyses.

Proteomic techniques can be divided to two groups [38]:

1. Discovery proteomics: “unbiased” or “hypothesis-free” methods where the target protein(s) is unidentified and the focus is on global protein profiling to identify differences between samples. These methods are particularly useful tools for biomarker discovery when potentially thousands of unknown proteins are to be investigated. Two-dimensional poly-acrylamide gel electrophoresis (2DE) and liquid chromatography (LC) are amongst the common tools used in separation of a large amount of proteins followed by mass spectrometry- (MS-) based techniques for relative quantification of proteins
2. “Targeted” techniques where the protein(s) of interest is already known. These methods commonly use sensitive antibodies with high affinity for target proteins. They include protein or tissue microarray and western blotting (WB) techniques. High-throughput LC-MS methods include selected reaction monitoring (SRM) of a predefined series of precursor/product ion pairs, termed transitions, that are specific to the protein analyte. When SRM is applied to investigate multiple transitions, this method is referred to as multiple reaction monitoring (MRM). A further development of parallel reaction monitoring (PRM) can also be utilised as a targeted technique, especially for confirmatory data, which provided alternatives to traditional antibody-based methods (Table 1).

The utility of quantitative proteomics, discovery, and targeting, for diagnosis and therapy of human disease, is reviewed by Cifani and Kentsis [45].

In this review, we have endeavoured to collate all published studies on IBD incorporating newer high-throughput proteomic techniques, through comprehensive electronic search using “PubMed” website search from 2000 to 2018, with extra ad hoc reports from older seminal papers in the subject. The search criteria included combination of “proteomics,” “mass spectrometry,” and/or “protein array” with “IBD,” “UC,” “Crohn's disease,” “Crohn's colitis,” and/or “colitis” (last accessed 29th September 2018).

## 4. Research in IBD

### 4.1. Biofluid Proteomics in Inflammatory Bowel Disease

Biofluids and excrements including faeces, plasma, ascites fluid, urine, and saliva allow convenient sampling modalities for biomarkers and patient screening that are indicative of underlying immunopathophysiologic changes and therefore are important sources of interest for proteomic analysis [44].

Plasma proteomics comprises identification, quantification, and further investigation of plasma/serum proteome in terms of their functions and interactions, both in health and in disease. It therefore encompasses investigation of secretome, exosome, and cellular proteins released as part of apoptotic vesicles. Its relative ease of sample collection and corresponding representation of pathophysiological changes in humans have made plasma proteomics a rapidly expanding area of interest in biomarker discovery, recommended for its stability relative to serum by the Human Proteome Organization [46]. Studies in IBD have focused on serum proteomics which represents a different form of the blood sample to plasma: serum is derived from blood that has coagulated, fibrin clots blood cells, and related coagulation factors are separated from serum by centrifugation. For plasma analysis, an anticoagulant such as EDTA or heparin is added before the removal of blood cells. The presence of fibrinogen and other clotting factors in plasma results in a higher protein concentration than serum [47]. These differences in the sample matrix require consideration when performing biomarker discovery study or evaluating the clinical use of biomarkers [48]. Complete profiling of such complex biological samples is still unachievable due to the presence of a high concentration of few proteins and requires depletion; in particular, albumin, immunoglobulins, serotransferrin, and haptoglobin represent more than 99% of the total serum and plasma proteins and interfere with the detection of less abundant proteins, limiting the analytical efficiency of LC-MS/MS. Therefore, their removal is essential to making low-abundant species detectable; a range of immunoaffinity reagents are available, as reviewed by Pisanu et al. [49].

### 4.2. Human Studies on Biofluids

Meuwis et al. investigated the sera of patients with IBD, healthy controls, and inflammatory controls (other than IBD) in order to compare the proteome profile specific for each condition. Using surface enhanced laser desorption ionization-time of flight-mass spectrometer (SELDI-TOF-MS) technology [50], they successfully purified and identified four acute-phase reactant proteins as potential biomarkers of disease activity including platelet factor 4 (PF4), haptoglobin *α*2 (Hp*α*2), fibrinogen-*α* chain (FIBA), and myeloid-related protein 8 (MRP8), two of which (PF4 and Hp*α*2) were further confirmed through standard methods. These are all known proteins of acute-phase inflammation, but their differential distribution amongst patient groups could help to discriminate IBD from controls [50]. PF4 belongs to the CXCL chemokine family also known as chemokine (C-X-C motif) ligand 4 (CXCL4), produced mainly by megakaryocytes and released at the injury site when activated [51–53]. Haptoglobin (Hp) is a multimeric serum protein and is generally used as a marker of inflammation and haemolysis; the molecule consists of 2*α* and 2*β* subunits connected by disulphide bridges. Two haplotypes of Hp*α* (*α*1 and *α*2) exist, which give rise to phenotypes Hp*α* 1-1, Hp*α* 1-2, and Hp*α* 2-2. Meuwis et al. demonstrate that the Hp*α*2 subunit is independent of Hp*α*1 and *β* suggesting this as a new biomarker specific for IBD [50]. Further work by Vanuytsel et al. identified human zonulin (an important physiological modulator of paracellular intestinal permeability) as prehaptoglobin-2 (pre-HP*α*2), a protein which is previously regarded as simply the inactive precursor for HP*α*2 [54]. Allele HP*α*2 and genotype HP*α* 2-2 have been shown to be overrepresented in different immune diseases like rheumatoid arthritis (RA), systemic lupus erythematosus (SLE), and type 1 diabetes and in patients with IBD relative to healthy controls [54–58]. MRP8 and MRP14 are two abundant cytoplasmic proteins of phagocytes that function as endogenous activators of Toll-like receptor 4 and thereby amplify phagocyte activation during inflammation upstream of TNF-*α*–dependent effects [59]. MRP8, which is the active component of the MRP8-MRP14 complex, induces intracellular translocation of myeloid differentiation primary response protein 88 and activation of interleukin-1 receptor–associated kinase-1 and NF-*κ*B, resulting in an elevated expression of TNF-*α* [59]. MRP8 is also part of the calprotectin complex, a biomarker of clinical endoscopic and disease activity in IBD when quantified in the stool [60].

In order to assess responders and nonresponders to infliximab therapy in CD before and after the treatment, Meuwis et al. exploited serum proteomic profiling using SELDI-TOF-MS technology in a subsequent study, which showed a positive correlation between PF4 and nonresponse to infliximab therapy in a post hoc analysis [61].

Kanmura et al. investigated the sera isolated from patients with UC (both active and inactive), CD, colon cancer, infectious colitis, and normal control in an attempt to discover biomarkers of disease activity specific to each disease state. They employed protein array technology by coupling the ProteinChip system with SELDI-TOF/MS for serum protein profiling [62]. They identified that 3 increased in abundance proteins, namely, human neutrophil peptides 1-3 (HNP 1-3) (antimicrobial peptides from *α*-defensin groups of proteins secreted by neutrophils and phagocytes), in the serum of active UC patients in comparison to the rest of the patients' subgroups including UC in remission, CD, infectious colitis, and healthy controls. They further confirmed these data using enzyme-linked immunosorbent assays (ELISA) and proposed HNP 1-3 as new biomarkers of UC disease activity [62].

To investigate biomarkers specific for children with IBD, Vaiopoulou et al. compared sera of adults and children with CD using 2DE followed by MALDI-TOF-MS and further validation by western blotting [63]. Ceruloplasmin (the acute-phase plasma protein produced by activated macrophages is a ferroxidase enzyme with bacteriocidal activity [64]) and apolipoprotein B-100 (APOB) were significantly overexpressed in children, whereas clusterin (a glycoprotein with antiapoptosis effect) [65] was found to be significantly overexpressed in adults with CD [63]. They further confirmed the differential expression of ceruloplasmin and clusterin with western blotting. Overexpression of clusterin may reflect the age-related change in the level of this protein irrespective of CD pathogenesis, as shown by Ignjatovic et al. [66]. The increase in abundance of APOB in this study contradicts a previous report by Koutroubakis et al., which showed a lower level of this protein in serum from a group of adult patients with CD relative to controls [67]. Whether this new finding in a child population suggests an alternative activation pathway is unknown, although the IL-6 stimulatory effect on the level of APOB mRNA levels [68] and its elevated serum levels in the adult and child population with IBD [69, 70] may explain overexpression of APOB in this study.

Hatsugai et al. investigated peripheral blood mononuclear cell proteins from a cohort of 17 UC and 13 CD patients using 2DE and discriminant analysis (orthogonal partial least squares-discriminant analysis or OPLS-DA) with subsequent quantitative MALDI-TOF MS/MS analysis in a search for a differential proteome signature between UC and CD [71]. They identified a model of 58 protein spots, which could discriminate between UC and CD with good performance for differentiation (*R*^2^ = 0.948) and prediction of the diseases (*Q*^2^ = 0.556). They further successfully identified 11 out of 58 protein spots by MS/MS analysis, which were assigned to seven kinds of proteins including Annexin A6 (ANXA6), peroxiredoxin 2 (PRDX2), a/b-hydrolase domain-containing protein 14B (ABHD14B), cyclophilin A (peptidyl isomerase A, PPIA), carbonic anhydrase 2 (CA2), beta actin (ACTB), and protein S100A9 (S100A9). ANXA6, PRDX2, ABHD14B, and PPIA were increase in the UC patients compared to the CD patients, of which the increase of PRDX2, ABHD14B, and PPIA was statistically significant. In contrast, CA2, ACTB, and S100A9 were found to be decreased in abundance in the UC patients compared to the CD patients, of which only the decrease in ACTB was statistically significant [71]. Four of the identified proteins were found to be associated with inflammation and oxidation/reduction (PPIA, S100A9, PRDX2, and CA2), and the remaining three were associated with the formation of the cytoskeleton (ACTB), endocytotic trafficking (ANXA6), and activation of transcription (ABHD14B). They did not match their protein spot analysis for different stages of UC/CD disease activity from the outset; hence, their proposed model based on a wide disease activity index range (SCCAI range 4-15 for UC and IOIBD score range 0-6 for CD) makes this discriminatory tool less predictable and reproducible.

Gazouli et al. investigated differential serum protein levels corresponding to the degree of infliximab responsiveness in a small group of CD patients using 2DE and MALDI-TOF-MS proteomic methods [72]. They identified a panel of increased-in-abundance proteins in the primary infliximab nonresponsive group including apolipoprotein A-I (APOA1), apolipoprotein E (APOE), complement C4-B (CO4B), plasminogen (PLMN), serotransferrin (TRFE), beta-2-glycoprotein 1 (APOH), and clusterin (CLUS) in comparison to the infliximab-induced remission group. They further confirmed CLUS and APOA1 overexpression in the primary nonresponder group by WB. Moreover, leucine-rich alpha-2-glycoprotein (A2GL), vitamin D-binding protein (VTDB), alpha-1B-glycoprotein (A1BG), and complement C1r subcomponent (C1R) were significantly increased in abundance in the serum of the infliximab-induced remission group as compared to the baseline pre-infliximab treatment serum [72]. It should be noted that this was a single-centre study with limited patient number and no further confirmation of the results in an independent group of patients.

In order to evaluate the specific changes in serum peptide abundance, Nanni et al. fractionated low molecular weight (LMW) serum proteins with a 10 kDa cut-off from patients with clinically active CD (*n* = 15; CDAI > 150) and healthy controls (*n* = 48) and utilised a reversed-phase nano-LC ESI/Q-TOF MS approach combined with targeted MS/MS analysis [73]. Fibrinopeptide A (FPA) (a peptide released from fibrinogen during clotting of the blood), peptides from complement 3 protein (C3) and its fragment (C3F), and peptides from apolipoprotein A-IV were identified as the peptides whose concentration mostly increases in CD in this study. Previous studies have demonstrated the anti-inflammatory effect of apolipoprotein A-IV in DSS-induced murine colitis in vivo [74] and considered it as an independent predictor of disease activity in patients with IBD [75]. Although the differential LC–MS analysis was performed only on the LMW proteome enriched during the preanalytical step, all the identified proteins in the study had a MW much higher than 10 kDa [73]. Therefore, they proposed a specific exoprotease activity in CD that involves procoagulant peptides as substrates (fibrinopeptide A) and postulated that platelet-derived microparticles (PDMPs) may contain yet unidentified exopeptidase enzymes with proinflammatory properties [73].

### 4.3. Animal Studies on Biofluids

In a murine model of IBD using interleukin-10 knockout (IL-10^−/−^) mouse, Viennois et al. performed a longitudinal study of circulating serum proteins using 2D GE and MALDI-TOF/TOF MS [76]. Female IL-10^−/−^ mice were monitored for colitis development for 15 weeks: at weaning (day 30) (no colitis), 15 weeks post-weaning (day 135) (severe colitis), and at an intermediate time point (day 93) (mild colitis). They identified and further confirmed (via WB or ELISA) a panel of 12 protein biomarkers, which could differentiate serum samples in mid- to late-stage IL-10^−/−^ mice from early noninflamed IL-10^−/−^ mice. The proteins were involved in a range of biological processes such as gene expression, glycosylation, signal transduction, metabolism, cell differentiation, translation, phosphorylation, immune response, protein denaturation, aging, and menopause. Of note, alpha-1-B glycoprotein (A1BG) was also decreased, whereas haptoglobin (HP), pregnancy zone protein (PZP), and hemopexin (HPX) were increased throughout the colitis progression in IL-10^−/−^ mice. Peroxiredoxin 2 (PRDX2) was transiently increased during mild colitis development, whereas transferrin (TF) was only increased later in severe colitis mice. In order to compare the IL-10^−/−^ chronic colitis model to a model of acute colitis, the above confirmed biomarkers were trialled in a DSS mouse model of acute colitis; PZP and PRDX2, previously found to be increased in colitic IL-10^−/−^ mice, were not altered in mice treated with DSS, suggesting that these markers are specific to a chronic inflammatory state. A1BG was observed to be decreased after DSS treatment, and HP and HPX were drastically increased, showing that A1BG, HP, and HPX were altered in the same way as was previously observed in colitic IL-10^−/−^ mice. To investigate the specificity of these biomarkers to intestinal inflammation, they further explored these proteins in a model of extraintestinal inflammation with collagen antibody-induced arthritis (CAIA). The overall outcome showed that PZP, COL1A1, and PRDX2 were specific to intestinal inflammation development with PZP and PRDX2 being specific to the IL-10^−/−^ model. The serum amyloid P-component and transthyretin were specific to the development of arthritic inflammation, whereas HP, inter-alpha-trypsin inhibitor heavy chain 4 (ITIH4), HPX, complement component 3 (C3), and A1BG were found to have an altered level in any inflammatory conditions.

### 4.4. Tissue (Cell) Proteomics in Inflammatory Bowel Disease

The application of cellular proteomics in IBD has been investigated in several studies using surgical or endoscopic colonic tissue biopsies, as well as cultured colonic cell lines [77].

### 4.5. Human Studies Using Tissues

Distinguishing UC and Crohn's colitis in clinical settings may be important as this may affect the medical and surgical management plan - particularly when considering panproctocolectomy and ileo-anal pouch surgery in a colitic patient. The differentiation can be very difficult despite all available clinical, radiological, endoscopic, and histological criteria; consequently, about 15% of colitic patients are being labeled as “indeterminate colitis” due to inconclusive investigation outcomes and change of diagnosis from UC to Crohn's colitis occurs in another 15% following colectomy [78].

In an attempt to discover a more sensitive differentiating diagnostic tool between Crohn's colitis (CC) and UC, M'Koma et al. investigated the ability of histology-directed MALDI-MS to determine the proteomic patterns between each group in a pilot study [78]. They compared uninflamed versus inflamed submucosa in UC and CC groups separately using MALDI-MS to determine whether this method can distinguish inflammation in each group. They then compared the proteomic outcomes from inflamed submucosa in UC and CC, uninflamed submucosa in UC and CC, and inflamed mucosa in the two groups to determine whether this method could distinguish UC and CC. They utilised a histology-directed protein profiling previously described [79] in order to separate inflamed and uninflamed areas in mucosa and submucosa accordingly. Pairwise comparative analyses of the clinical groups obtained from MALDI-MS (comparing the discrete protein peaks in each group) were able to distinguish CC and UC specimens by profiling the colonic submucosa only [78]. They did not identify the potential discriminatory proteins in MS spectral peaks. Furthermore, the small sample size of 51 patients (*n* = 24 CC and *n* = 27 UC) made the estimation of statistical significance of the intergroup differences approximate. Using the same histology-directed MALDI-MS methodology, the group recently analysed histologic layers of 62 confirmed IBD tissues (26 from CC and 36 from UC) in order to distinguish between UC and CC [80]. A total of 1257 spectra from 62 total IBD cases were used for statistical analysis of the mucosa. Out of 312 total peaks in the averaged spectra, 114 were found to have Wilcoxon rank-sum *p* values of less than 0.05 [80]. A support vector machine algorithm consisting of 25 peaks was able to discriminate spectra from CC and UC with 76.9% spectral accuracy when using a leave-20%-out confirmation of the results. The application of the model to the entire dataset resulted in an accurate classification of 19/26 CC patients and 36/36 UC patients when using a 2/3 correct cut-off [80]. Whether this discriminatory methodology can be used to differentiate UC from CC in patients with indeterminate colitis needs further investigation, as none of the patients recruited in this study previously had an equivocal diagnosis.

In a pilot study by Shkoda et al., IEC purified from colonic and ileal regions of surgically resected tissues in UC (*n* = 6) and CD (*n* = 6) were compared to normal-looking tissue from colon cancer patients (*n* = 6). 2D SDS-PAGE analysis was combined with peptide mass fingerprinting via MALDI-TOF MS. Amongst the proteins found with differential expression in both UC and CD groups, statistical significance was reached for 4 proteins including L-lactate dehydrogenase, carbonyl reductase (NADPH prostaglandin E2 reductase), keratin 19, and the Rho-GDP dissociation inhibitor R (Rho GDI *α*) [81]. Western blotting further confirmed the MS data regarding induced expression of Rho GDI *α* in inflamed mucosa from both patient groups in comparison to control, but this overexpression was also observed in the mucosa from patients with sigmoid diverticulitis suggesting that this latter change in Rho GDI *α* overexpression is probably due to inflammation rather than the IBD-specific process [81]. They further compared inflamed colonic mucosa to the noninflamed mucosa in two separate UC patients. Although there was a significant variation between the number of differentially expressed proteins (28 versus 3), the most significant changes in protein expression were detected for Annexin A2 and programmed cell death protein 8 in these two patients [81]. Lactate dehydrogenase is a cellular tissue enzyme that catalyses the conversion of pyruvate (the end product of glycolysis) to lactate in hypoxic states and hence is a general marker of tissue injury and damage. Increased carbonyl reductase (an oxidoreductase enzyme utilising NADP/NAD as cofactor) expression is also suggestive of increased oxidative burst in the colonic mucosa during inflammation [82]. Intermediate filaments (IF) are one of the main components of the human cell cytoskeleton, which mainly consist of keratins. Keratins 8, 18, and 19 constitute the main keratins in the intestinal epithelial cells. Our group has recently shown a reduced level of keratins 8, 18, and 19 in the insoluble fraction of active colonic mucosa of UC patients in comparison to proximal inactive mucosa from the same patients as well as normal controls [83–85]. Rho GDI (a modulator of Rho-GTPase family of enzymes [86]) complexed with RhoA has recently been shown to be involved in NF-*κ*B activation cascade [87]. Annexin A2 is a pleiotropic protein from the calcium-dependent phospholipid-binding protein family that orchestrates a growing spectrum of biologic processes including involvement in cellular growth and in signal transduction pathways [88]. In a recent study by Zhang et al. published in Chinese language (the abstract is available in English), Annexin A2 expression has been shown to be upregulated in inflamed UC mucosa in comparison to normal control using the immunohistochemistry technique followed by Q-PCR to confirm mRNA overexpression of Annexin A2 [89]. Programmed cell death protein 8, otherwise known as caspase 8, has been shown to control necroptosis of Paneth cells and potentially the death of intestinal epithelial cells in patients with Crohn's disease and hence to participate in the mucosal inflammation process [90]. The use of normal-looking colon tissues from colon cancer patients as a control group may cause potential spurious results considering the previous study by Polley et al., which indicated that protein expression in the apparently normal colonic mucosal field is modified in individuals with neoplastic lesions at sites distant from the lesion, indicative of a field effect [91].

In order to explore the protein profile in UC, Fogt et al. deployed proteomic analysis (2D PAGE followed by LC-MS/MS) on surgically resected colonic tissue from 5 patients with active UC comparing active resected mucosa/submucosa to the uninvolved grossly normal mucosa of the UC patients [92]. Increased abundance of proteins in the active UC tissues that are mainly involved in inflammation and repair included protocadherins (a subtype of the cadherin superfamily of proteins involved in cell adhesion and the cell-cell interaction process. These findings were linked to the retention of the monolayer morphology of proliferating cells [93, 94]), *α*-1 antitrypsin (a protease inhibitor and acute-phase response protein [95]), tetratricopeptide repeat domains (associated with heat shock proteins [96]), caldesmon (a calmodulin-binding protein; calmodulin is in turn a regulator of gap junction channels [97]), and mutant desmin. The presence of mutated desmin (a type III intermediate filament) was confirmed by western immunoblotting. The authors hypothesized the possibility that desmin mutation may be a primary or secondary feature in the pathogenesis of UC, although it could also represent a random mutation in a high-turnover cell population [92]. In a recent genetic analysis study by Avitzur et al., tetratricopeptide repeat domain 7A mutations have been linked to very early onset IBD as a result of severe apoptotic enterocolitis due to defects in enterocytes and T cells [98].

Hsieh et al. used 2D PAGE and MALDI-TOF-MS for peptide mass fingerprinting (PMF) analysis in colonic tissue biopsies from 4 patients with active chronic UC, 3 patients with infectious colitis, and 5 normal individuals to explore differentially expressed proteins in UC [99]. They identified 19 distinct proteins including 13 decreased-in-abundance and 6 increased-in-abundance proteins in the active compartment of the UC mucosa. Of the 13 decreased-in-abundance proteins in the active UC colonic mucosa, eight were either the mitochondrial component proteins or proteins directly involved in oxidation/phosphorylation [99]. These results were confirmed using immunoblotting analyses and immunohistochemical (IHC) studies. They further compared active distal UC mucosa with proximal normal-looking inactive mucosa in two patients using similar proteomic analysis and observed relatively similar protein expression patterns between inactive UC mucosa and normal colonic mucosa except for downregulation of prohibitin (PHB) (the major component protein of the mitochondrial inner membrane) in inactive UC mucosa similar to the active distal UC compartment. The authors hypothesized the pivotal role of mitochondrial dysfunction in the pathogenesis of UC [99]. PHBs are members of a highly conserved protein family that are found in multiple cellular compartments including the mitochondria, nucleus, and the plasma membrane [100]. Nuclear and mitochondrial PHBs are thought to be involved in cellular differentiation, antiproliferation, morphogenesis, and cellular survival (through the Ras-Raf-MEK-Erk pathway) and in particular maintenance of the functional integrity of the mitochondria and protection of cells from various stresses [100]. Using a yeast two-hybrid method, Bacher et al. identified PHB as a binding partner for Annexin A2 and *α*-actinin, which further attribute their interactions with members of the cytoskeletal proteins [101].

In order to characterise the differential proteins associated with inflamed mucosa in active UC, Poulsen et al. investigated tissue biopsies from active distal UC patients using 2DE and MALDI-TOF MS technology [102]. They compared the whole lysate extract isolated from active rectal UC mucosa and reciprocal inactive proximal mucosa from the same patient groups and identified 222 statistically significant protein spots using image analysis. Protein profiles from different colonic regions of normal control individuals were compared to identify proteomic changes reflecting inflammatory status of the colon rather than position specific effects. The data showed a generally similar proteomic signature in rectum and left colon flexure except for alpha-enolase and hydroxymethylglutaryl–CoA synthase, which had differential protein levels between rectum and left colon flexure in both patients and normal control individuals suggesting that the two latter proteins are proteins associated to segment sampling rather than potential disease markers [102]. Principal component analysis (PCA) grouped noninflamed samples separately from the inflamed samples suggesting a distinctive proteomic signature of the colon mucosa in acute UC. A total of 43 individual protein spots were identified corresponding to 33 individual proteins. These proteins included those involved in energy metabolism (triosephosphate isomerase, glycerol-3-phosphate dehydrogenase, alpha enolase and L-lactate dehydrogenase B-chain, isocitrate dehydrogenase, inorganic pyrophosphatase, and enoyl-CoA hydratase) and in oxidative stress (superoxide dismutase, thioredoxins, and selenium-binding protein) [102]. The authors hypothesized that different protein profiles of several glycolytic enzymes and other proteins involved in energy may indicate inflammation-associated alterations in energy metabolism in UC patients, which could in turn be related to malfunction of the utilisation of n-butyrate (the preferred source of energy for colonocytes in the distal colon [103]). They further postulated that the observed lowered enoyl-CoA hydratase in inflamed tissue might infer impaired fatty acid oxidation; the state of energy deficiency is further strengthened by change in the glycolytic pathway reflected as a higher expression of glycolytic enzymes in inflamed mucosa [102].

In an attempt to find specific biomarkers in IBD including its subtypes, Han et al. investigated a small number of patients with active (*n* = 4) and inactive (*n* = 4) UC, active (*n* = 3) and inactive (*n* = 3) CD, and inflammatory polyps associated with UC (*n* = 2) as well as normal controls (*n* = 3) using label-free LC-MS analysis [104]. Bone marrow proteoglycan (PRG2), L-plastin 1 (LCP1), and proteasome activator subunit 1 (PSME1) were identified potential biomarkers for active CD. Following comparison to other published data, they further summarised certain proteins in active UC patients to be increased in abundance relative to normal controls. Candidate biomarkers were Tau tubulin kinase 2 (TTBK2), spectrin repeat containing nuclear envelope 2 (SYNE2), succinate CoA ligase 2, GDP-forming subunit (SUCLG2), and periostin (POSTN), as well as other proteins that are increased in both active UC and CD relative to normal tissues including fibrinogen, calprotectin (S100A8), myeloperoxidase (MPO), and neutrophil defensin-1 (DEFA1B and DEFA1) [104]. It is worth mentioning that the differentiation between active and inactive UC/CD in this study was based on clinical and endoscopic findings. The number of patients included in the study was another limitation of the study. PRG2 comprises the crystalloid core of the eosinophil granule, which has cytotoxic and microbicidal activities [105]. The protein is an enhancer of natural killer cell activity [106]. A preform of PRG2 also acts as a proteinase inhibitor, reducing the activity of pregnancy-associated plasma protein A (PAPPA) [107]. LCP1, one of three isoforms of the plastin protein family, is constitutively expressed at high levels in haemopoietic cell types as well as many types of solid tissue malignant cells [108, 109]. As a leukocyte-specific protein, LCP1 cross-links actin filaments into tight bundles, increasing the stability of actin-based structures such as podosomes and lamellipodia [110]. PSME1 is a multicatalytic proteinase complex that cleaves peptides in an ATP/ubiquitin-dependent process (ubiquitin-proteasome system) [111]. It may also harbour a regulatory role on the Rho GTPase activity [112]. Further investigation by Bennike et al. used a label-free (LFQ) proteomic analysis to compare UC to normal biopsies (*n* = 10, for each) and identified proteins with statistically significant altered abundance in the UC biopsies to be from neutrophils and associated with the formation of neutrophil extracellular traps (NETs) [113]. An increased abundance of neutrophils and the presence of neutrophil extracellular traps were confirmed by microscopy and the presence of extracellular DNA present in the UC colon tissue, suggesting stimulation of innate immunity [113], since NETS form part of the first line of immune defence [114].

In order to investigate differential proteomic changes in UC and tuberculosis colitis (TC), Kwon et al. examined colonic tissues from these two groups (*n* = 6 in each group) in comparison to normal controls (*n* = 6) using 2DE and MALDI-TOF/TOF MS technology [115]. Of the over 1000 proteins isolated, mutant *β*-actin, *α*-enolase, and Charcot-Leyden crystal proteins were found to be differently abundant in these colitides relative to normal controls. In particular, *α*-enolase was significantly overexpressed in TC compared with normal tissue, but underexpressed in UC relative to control, suggesting *α*-enolase as a candidate biomarker for differential diagnosis of TC and UC [115]. Previous studies have shown upregulation of *α*-enolase mRNA levels in IBD [116] and downregulation at the protein level in UC [117], which may in turn be partly related to overexpression of *α*-enolase reactive antibodies found in 10-50% of patients with UC [116, 118]. Although the study suggested recruiting patients with active colitis, severity of disease activity was not quantified. Moreover, there was no confirmation of the results of the findings on a new set of tissue samples in these cohorts.

To identify molecular signatures in UC, Zhao et al. compared intestinal mucosa from 12 UC patients with 12 normal controls using comparative proteomic analysis with 2DE and MALDI TOF/TOF MS [117]. A total of 26 unique differential proteins were identified, including 12 increased-in-abundance and 14 decreased-in-abundance in the UC group. A differential protein cluster, consisting of 11 proteins (including Cdc42, vimentin, Annexin A2, HSP90B1, LSP1, GRP78, and cathepsin D that were increased in abundance in UC; and HSF2, galectin-3, VDAC-1, and MAWBP that were decreased in abundance) involved in the p38 mitogen-activated protein kinase (MAPK) pathway was deduced and confirmed by western blot. Furthermore, three proteins elicited from the protein cluster (phosphorylated p38, MAWBP, and galectin-3) were analysed by IHC on 58 UC and 60 normal tissue samples. Increased expressions of p-p38 and decreased-in-abundance MAWBP and galectin-3 were detected in active UC compared to normal samples [117]; hence, they concluded that the P38 MAPK pathway might be involved in UC disease activity. This observation was consistent with data from a DSS-induced experimental colitis murine model [119]. Others have proposed that the regulation of TNF-alpha, a key mediator in the inflammatory process in IBD, is interconnected with MAPK pathways [120].

Brentnall et al. applied the quantitative proteomic technique using LC MS/MS and iTRAQ labelling to study the protein expression of UC neoplastic progression [121]. They compared the proteomes of both dysplastic and nondysplastic mucosa from UC patients with high-grade dysplasia or cancer (UC progressors; *n* = 5 in each) to the colonic mucosa of UC patients who were dysplasia-free (UC nonprogressors; *n* = 5) and normal non-UC colon (*n* = 5) in order to investigate the possibility of field defect at the proteome level. They identified differential protein expression relating to mitochondria; oxidative activity and calcium-binding proteins are associated with UC neoplastic progression. Network analysis discovered that Sp1 and c-myc proteins might play roles in UC early and late stages of neoplastic progression, respectively. Two increased in abundance mitochondrial proteins in the UC progressor groups, namely, CPS1 and S100P, which were further confirmed by IHC experiment on 17 tissue samples. The S100 proteins are a multi-gene calcium-binding family comprising 20 known human members with a broad range of intracellular and extracellular functions, including regulation of protein phosphorylation and enzyme activity, calcium homeostasis, regulation of cytoskeletal components, and regulation of transcriptional factors [122]. A previous study by Fuentes et al. suggested that S100P is overexpressed in colon cancer relative to normal tissue and that S100P stimulates colon cancer cell growth, migration, Erk phosphorylation, and NF-*κ*B activation in vitro [123]. CPS1 is a mitochondrial enzyme involved in the urea cycle and expressed mainly in intestinal epithelial and liver cells; it detoxifies ammonia and, together with other enzymes of the urea cycle, is the de novo source of arginine [121]. Variations in the supply of arginine, due to alterations in urea cycle function, affect the production of nitric oxide [124]. Nitric oxide, in turn, can cause DNA damage and laxity in DNA repair and is associated with inflammation-associated cancers [125]. In this study, they proposed that the proteome of the nondysplastic mucosa from the progressor group was more akin to the proteome of high-grade dysplasia than it was to the nondysplastic mucosa of the nonprogressor group and concluded that there are changes in protein expression early in the neoplastic progression, before the histologic changes become evident in the epithelial cells.

In the search for objective biomarkers of dysplasia in UC, the collaborators from the same group (May et al.) investigated individual rectal and colonic epithelial fractions from UC patients with dysplasia or cancer (UC progressors) compared to similar samples from patients with no dysplasia/cancer (UC nonprogressors) in a label-free MS experiment coupled with HPLC [126]. Mitochondrial proteins, cytoskeletal proteins, RAS superfamily, proteins relating to apoptosis, and metabolism were the important protein pathways differentially expressed in the nondysplastic and dysplastic tissues of UC progressors, suggesting their importance in UC neoplastic progression. They further confirmed two differentially expressed mitochondrial proteins including TNF receptor-associated protein 1 (TRAP1), which displayed increased IHC staining in UC progressors (in both dysplastic and nondysplastic tissue), and CPS1, which showed a statistically significant difference in IHC staining between the nonprogressor and progressor groups [126]. TRAP1 is a mitochondrial heat shock protein involved in protection against oxidative stress and apoptosis. Previous studies have also shown increased expression of TRAP1 in colorectal carcinoma [127].

In order to identify disease-specific autoantigens in CD, Zhou et al. applied immunoproteomic techniques using a combination of 2D PAGE, WB, and MALDI MS on surgically obtained mucosal lesions from 8 active CD patients [128]. Mixed serum samples from the same CD patients were used as a primary antibody source for detection of autoantigens in the colonic tissue. The adjacent normal-looking mucosa on each patient was used as a control to identify increased protein spot intensities on each gel. They identified 6 differentially expressed protein spots including prohibitin, calreticulin, apolipoprotein A-I, intelectin-1, protein disulphide isomerase, and glutathione s-transferase pi. Prohibitin and glutathione s-transferase overexpression in the mucosal lesion of active CD patients relative to the neighbouring normal mucosa was further confirmed with WB on 4 separate CD patients [128]. A small number of CD patients together with lack of controlled patients and follow-up data were the main limitations of this study.

### 4.6. Cell Line Studies

Araki et al. compared UC-associated cancer and sporadic colon cancer cell lines using proteomic analysis with 2D GE and LC-MS/MS in the search for differentially expressed proteins in the two groups and found a higher expression of heat shock protein of 47 kDa (HSP47) in UC-associated cancer cell lines [129]. IHC validation of the result on 63 UC-associated lesions and 81 sporadic colon tumour lesions confirmed significantly higher levels of HSP47 in UC-associated colon cancers than in sporadic counterparts, the expression increasing with a progression of neoplastic lesions. HSP47 coexpression with type I collagen in the cytoplasm was further confirmed by double immunofluorescence staining of cell lines [129]. HSP47 is a collagen-binding, stress-inducible protein localised in the ER that participates in intracellular processing, folding, assembly, and secretion of procollagens, especially in collagen-secreting cells, such as fibroblasts [130]. In tissue, although HSP47 was found to be expressed in both epithelial and stromal cells within cancers, only the former showed an increase with progression to greater malignancy [129].

Barceló-Batllori et al. evaluated homogenates from DLD-1 colorectal adenocarcinoma cells (as in vitro IBD model) before and after exposure to IFN-*γ*, IL-6, and IL-1 and utilised 2D PAGE together with MALDI-TOF-MS in a search for cytokine-regulated proteins in intestinal epithelial cells (IEC) in IBD [131]. Amongst several identified proteins in the in vitro model (including tryptophanyl-tRNA synthetase, indoleamine 2,3-dioxygenase, heterogeneous nuclear ribonucleoprotein JKTBP, interferon-induced 35 kDa protein, proteasome subunit LMP2, and argininosuccinate synthetase), overexpression of indoleamine-2,3-dioxygenase (IDO) was confirmed in the purified IEC from IBD patients in comparison to normal mucosa [131]. Indoleamine-2,3-dioxygenase-1 (IDO1), which is well-characterised in the gut, is the first and rate-limiting step in tryptophan catabolism along the kynurenine pathway [132]. IDO expression has been linked with immune tolerance at the maternal-foetal interface via suppression of T cell proliferation by seminal work of Munn et al. [133, 134]. Matteoli et al. later observed that CD103^+^ gut dendritic cells expressing IDO1 support regulatory T-cell conversion while suppressing Th1/Th17 differentiation to promote oral tolerance and limit gut inflammation [135]. IDO1 expression is stimulated by inflammatory cytokines including IFN-*γ*, TNF-*α*, and IL-1B [132]. Consistent with this, IDO1 is one of the highly upregulated genes in human IBD [136].

### 4.7. Animal Models

Liu et al. studied alteration of lymphocytic protein profiles in a rat model of 2,4,6-trinitrobenzene sulfonic acid- (TNBS-) induced colitis [137]. After extracting proteins from the harvested lymphocytes 7 days post-TNBS/ethanol colitis induction, they compared protein spots on 2D PAGE in lymphocytes from colitic and control rats using PDQuest 2D image analysis followed by identification of differentially expressed protein spots using MALDI-TOF-MS. The identified differentially expressed proteins were confirmed with reverse-transcription polymerase chain reaction (RT-PCR). There were 26 proteins that altered in abundance of which 17 and 9 were increased or decreased in abundance, respectively. Proteins involved in the regulation of the cell cycle, cell proliferation, and signaling transduction were increased (NDPKs and E2N), and the one involved in inflammation (MRP14) was increased. Altered proteins include those associated with apoptosis (increased-in-abundance IL-12 p40, decreased-in-abundance proteasome activator complex subunit 2, CARD, and PYD domain-containing protein) and metabolic/oxidative stress response (increased-in-abundance ATP-citrate synthase, phosphoglycerate mutase, and dismutase) activities, as well as others [137]. Nucleoside-diphosphate kinase (NDPK) catalyses the phosphorylation of nucleoside diphosphate to nucleoside triphosphate; it confers protection from cell death by Bax and alters the cellular levels of several antioxidant enzymes including Gpx5 [137, 138]. Ubiquitin-conjugating enzyme (E2N) plays a role in the error-free DNA repair pathway and contributes to the survival of cells after DNA damage [137]. Its action has been linked to the regulation of activating transcription factor 2 (ATF2) and NF-kappa B transcriptional activator precursor p105, which are in turn essential players in lymphocyte activation [139, 140]. Myeloid-related protein- (MRP-) 8 (S100A8) and MRP14 (S100A9) are members of the S100 family of calcium-modulated proteins that regulate myeloid cell function and control inflammation [141]. Proteasome activator complex subunit 2 is required for immunoproteasome assembly and efficient antigen processing [137]. IL-12 p40 is a proinflammatory cytokine with an important role in innate and adaptive immunity [142, 143]. The protein encoded by the IL-12B gene, which is a susceptibility locus in CD and UC [144, 145]. Caspase activation and recruitment domain- (CARD-) and pyrin domain- (PYD-) containing proteins promote caspase-mediated apoptosis predominantly through the activation of caspase 9 [137]. The key structural scaffold for inflammasome (plays a role in host defence against microbial pathogens and gut homeostasis) assembly is composed of filaments of PYD and CARD in the sensor, adaptor, and effector components [146]. Dismutase delivers copper to copper-zinc superoxide dismutase [137].

The decrease of proapoptosis proteins and the increase of antiapoptosis proteins shown in this study may contribute to the inhibition of the lymphocyte apoptosis, which subsequently result in the overactivation of lymphocytes in colitis rats. Lymphocyte homeostasis/apoptosis is pivotal in orchestrating efficient immune responses and avoiding autoimmunity [147]. Liu et al. raised concerns about some limitations in the study including difficulty analyzing some membrane proteins and low-abundance proteins by 2D PAGE, as well as poor reproducibility and variations of spot intensity observed with silver staining as previously described [148].

In a murine model of CD using multidrug resistance-targeted mutation (Mdr1a^−/−^) mice, Barnett et al. investigated the effect of polyphenol-rich green tea extract (GrTP) on the inflamed mouse colon transcriptome and proteome [149]. Transcriptomic data (whole genome microarray) and the proteomic analysis 2DE/LC-MS technique were performed in parallel on the same samples. Integration of the data show reduced abundance of transcripts and proteins associated with immune and inflammatory response as well as fibrinogenesis pathways and increased abundance of those associated with xenobiotic metabolism pathways in response to GrTP [149]. Increased abundance of peroxisome proliferator-activated receptor-*α* (PPAR-*α*) and decrease in signal transducer and activator of transcription 1 (STAT-1) protein abundance appeared to be the two key modulatory effects in response to GrTP in Mdr1a^−/−^ mice. Dietary intake of polyphenols derived from green tea was demonstrated to ameliorate intestinal inflammation in the colon of a mouse model of CD and therefore may play a part in an overall IBD treatment regimen [149]. PPARs are ligand-activated transcription factors of the nuclear hormone receptor superfamily [150]. PPARs play essential roles in the regulation of cellular proliferation and differentiation, atherosclerosis, inflammation, metabolism, infertility, tumourigenesis, and demyelination [151–153]. PPAR-*α* is expressed at high levels in organs with significant catabolism of fatty acids and is also shown to have a role in controlling colonic inflammation and mucosal tissue homeostasis [150, 154, 155]. In addition to the important role of NF-*κ*B and MAPK inflammatory pathways in IBD, many cytokines exert their intracellular signalling by activating the JAK/STAT pathway [156]. Overexpression and activation of STAT1 have previously been linked to IBD relapse [157]. The beneficial effects of GrTP in this study could be due to the specific anti-inflammatory effect of the compound on this particular genetic defect of IBD mouse model (Mdr1a^−/−^).

### 4.8. Organelle Proteomics (Subproteomics) in Inflammatory Bowel Disease

Complexity and abundance of proteins with very variable concentrations and dynamic range have a significant impact in the sensitivity of cellular proteomic methodologies [77]. Subcellular fractionation and proteomics (subproteomics) are an ideal adjunct for reducing cellular sample complexity; it can combine current proteomic methodology for enrichment and analysis of intracellular organelles and low-abundance multiproteins [158].

Subcellular fractionation involves disruption of the cellular assembly (homogenisation) followed by fractionation of the homogenate to different subgroups, based on their physical properties, by using differential centrifugation disruption; moreover, quality control methods including quantitative western blotting (targeting specific organelle-marker proteins) and qualitative/morphological analysis (electron microscopy) can ensure optimal subcellular fractionation of interest [158]. Although absolute subcellular compartment purification is not possible due to cofractionation of particles with similar physical characteristics, this method is a powerful adjunct to the proteomic analysis [158, 159].

### 4.9. Cell Line Studies on Organelles

One of the early reports of organelles proteomic application cells was by Fialka et al., who coupled subcellular fractionation with 2D PAGE, immunoblotting and microsequencing for investigation of endosomal proteome as method for analysis of epithelial cells [160].

### 4.10. Human Studies Using Subcellular Fractions

O'Morain et al. carried out subcellular fractionation on rectal biopsies taken from patients with UC, Crohn's colitis, and CD with no rectal involvement as well as normal controls by homogenizing samples in isotonic sucrose followed by sucrose density gradient centrifugation [161]. The gradient fractions and tissue homogenates were then assayed for marker enzymes for the plasma membrane (5′-nucleotidase), mitochondria (malate dehydrogenase), peroxisomes (catalase), cytosol (lactate dehydrogenase), lysosomes (N-acetyl-beta-glucosaminidase), and endoplasmic reticulum (neutral-alpha-glucosidase); they found selective and specific alterations in the principal subcellular organelles, especially the plasma membrane, lysosomes, and mitochondria in IBD [161].

In a comparative study exploring differential protein expressions in cellular compartments of IEC (nuclei, membranes, and cytosols) in CD patients versus control, Nanni et al. isolated epithelial cells from surgically resected colonic tissue using a previously confirmed method [162] and utilised mono-dimensional gel electrophoresis (1DGE) combined with label-free nano-LC ESI/QTOF MS followed by targeted MS/MS analysis [73]. Annexin A1 (a nuclei membrane protein regulating phospholipase A2 activity) and malate dehydrogenase (a mitochondrial and cytosolic enzyme involved in the citric acid cycle as well as malate-aspartate shuttle [74]) were decreased in abundance in CD patients in comparison to controls. Protein increases were identified in each of the subcellular fractions. Examples include ubiquitin, tryptase precursor, heat shock 70 kDa protein-5, and ATP synthase beta. Increased levels of ubiquitin in the nuclear fraction were proposed to reflect increased activity of IECs, consistent with increased levels of ATP synthase subunit beta in the membrane fraction [73]. Heat shock 70 kDa protein-5 is a multifunctional protein that participates in protein folding and calcium homeostasis and serves as an essential regulator of ER stress response [163]. This protein was increased in abundance in the membrane fraction of CD patients, as might be expected due to its role in the protection of the intestinal epithelium [73]. Tryptase (a serine protease also known as mast cell tryptase) has been shown to play an important role in acute DSS-induced colitis in mice [164].

Most of the proteins found in the study by Nanni et al. [155] were similar to those found in the earlier differential protein profiling study on IEC from IBD patients using 2D PAGE/MS methodology [81], thereby confirming these results. Heat shock proteins (Hsp) are considered chaperone proteins involved in the folding process of other cellular proteins [165]. Their expressions are increased in abundance in response to intracellular stresses including hyperthermia, ischemia/reperfusion, oxygen radicals, heavy metals, ethanol, and amino acid analogues [165]. Although upregulation of Hsp70 seems to be a natural response to inflammation as per Nanni et al., a previous study by Hu et al. contradicts this finding by showing that inducible Hsp expression (Hsp25/27 and Hsp70) is translationally (but not transcriptionally) decreased in abundance via phosphorylation and inactivation of eukaryotic initiation factor-2*α* (eIF-2*α*) in active IBD [166].

### 4.11. Secretome

Profiling and identification of active serine proteases secreted by the colonic mucosa of control and IBD patients was performed using a MS-based functional proteomic analysis to monitor availability of enzyme active sites in colonic tissues from CD or UC patients. The study employed activity-based probes, chemical proteomic tools, which possess a reactive group to mimic enzymatic substrate and covalently bind active proteases. Biotinylation of the ABP allows selective avidin-based affinity capture of the ABP-enzyme target, which is identified by MS and posttryptic digestion to generate peptides for LC-MS/MS analysis. Seven serine proteases including cathepsin G, plasma kallikrein, plasmin, tryptase, chymotrypsin-like elastase 3A, thrombin, and aminopeptidase B were identified, of which cathepsin G and thrombin were overactive in IBD patient samples compared to healthy controls [167]. These data are consistent with the earlier study by Dabek et al. reporting the increased presence of cathepsin-G in faeces of UC patients [168].

### 4.12. Intermediate Filaments

Intermediate filaments (IF) are one of the main components of the human cell cytoskeleton, which mainly consist of keratins (K). K8, K18, and K19 are the main keratins in the intestinal epithelial cells. Keratin alteration may play a role in the pathophysiology of ulcerative colitis (UC). K8^−/−^ mice develop chronic colitis via a mucosal chronic T-helper type 2 inflammatory response [169]. K8 and K18 play a role in TNF-*α*-induced apoptosis [170, 171]. A recent study by our group (Corfe et al.) exploited the subcellular fractionation technique to identify differential expression of intermediate filaments (IF) in UC patients at differing risk for colorectal cancer [172]. Colonic biopsies were collected endoscopically from the following patients: recent-onset colitis in remission (*n* = 8), longstanding pancolitis in remission (*n* = 10), and UC with PSC (*n* = 6). UC with dysplasia (both from the dysplastic lesion and distal rectal mucosa; *n* = 3), active distal UC (*n* = 10), proximal inactive mucosa from the same patients with active UC, and normal controls (*n* = 10) were also included. The IF fraction was prepared for quantitative analysis using iTRAQ. Significant differences in protein abundance were followed by immunoblotting and IHC. Acute inflammation resulted in reduced K8, K18, and K19 and vimentin (VIM) compared to controls and noninflamed proximal mucosa; reduced levels of IF–associated proteins were also seen in UC with PSC and UC with dysplasia (both from the lesion and remote distal rectal mucosa). Increased levels of K8, K18, and K19 and VIM in longstanding pancolitis in remission were noted relative to controls and recent-onset colitis in remission [172]. Increased expression of VIM, generally expressed in cells with a mesenchymal phenotype in longstanding pancolitis, may reflect morphological colonic tissue remodelling and architectural alterations, reflecting the chronic relapsing/remitting course of the disease as a consequence of accumulated damage during each active phase [173, 174]. We proposed a model for the pathogenesis of colitis-associated colorectal cancer (CAC) whereby acute inflammation reduces keratin levels and affects mucosal IF protein integrity which lags behind apparent clinical, microscopic, and endoscopic recovery; persistent failure of such recovery may be the cornerstone of pathogenesis of CAC [172]. It should be emphasised that this study was cross-sectional with limited patient number especially in the cancer risk groups (UC with PSC and dysplasia).

A brief description of each of the reviewed proteomic studies is provided in Table 2.

## 5. Conclusions

The human proteome surpasses the human genome by several orders of magnitudes with regard to complexity, dynamic range, and diagnostic capability [175]. New proteomic high-throughput technologies together with advances in bioinformatics science provide a great opportunity for exploring complex human conditions such as autoimmune disease and cancer. Despite many advances in understanding the IBD disease process over the past decade, mainly due to Human Genome Project and knockout gene techniques in animal model studies, the exact pathogenesis of IBD remains elusive.

The combination of a very large number of cellular proteins with highly variable concentration together with their susceptibility to enzymatic degradation and precipitation makes proteomics a challenging area of investigation. Nevertheless, careful sample selection, meticulous attention during fractionation of proteins, development of more specific subproteomic techniques to diminish the masking effect of high abundance proteins while enhancing the detection rate of low abundance ones, and fine-tuning of proteomic methodology together with future proteomic technology developments can improve protein uptake and reproducibility.

In summary, proteomic technologies combined with bioinformatics analysis are very useful yet underexploited tools in exploring complex disease processes such as IBD in terms of delineating its pathophysiology, as well as discovering key biomarkers of disease activity/remission, optimizing risk stratification for tailored individualized treatment, and revealing noninvasive predictors of evolution to CAC. Improved biomarkers remain an unmet need in inflammatory bowel disease (IBD) [176]. The use of biomarker panels has higher discriminatory ability than that of single protein markers. As an example, Starr et al. identified two biomarker panels for paediatric patients to differentiate ascending colon biopsies of CD from healthy control patients and UC from CD [177]. These biomarkers have recently been further confirmed in intestinal mucosal–luminal interface aspirates and stool. In terms of clinical translation, a key finding from this study is that the biomarkers demonstrate transferable results in stool samples providing a scope for noninvasive sampling for biomarker analysis samples [178]. Regulatory authorities have mandated the use of patient-reported outcomes for inclusion as an outcome measure in inflammatory bowel disease trials. However, as recently noted by Khanna et al., it is likely that CRP and FCP will continue the usage in clinical trials as a secondary end point or to stratify baseline disease severity; noninvasive biomarkers are yet to replace endoscopy review data [179]. In terms of personalised medicine, it is important to understand the translatability and variability of individual biomarkers for development of tools such as quantitative system pharmacology models to predict individual response [180]. Development in sample fractionation and MS technologies are providing step change to research in personalised medicine [181]. These include high-throughput fractionation systems with minimal handling and sample loss [182, 183]. Also, new MS developments such as BoxCar data acquisition [184] and parallel accumulation–serial fragmentation [185] can yield high coverage and dynamic range, for example, 90% coverage of the proteome of a human cancer cell line (>6200 proteins) [184, 185]. The biomarker field is however developing, but work is still required to translate biomarkers from proteomic analysis to clinical utility.

## 6. Future Perspectives

Depositing and mining data from the proteomic repositories such as the PRoteomics IDEntifications (PRIDE) database and archive has the potential to enable integration of interrelated IBD studies, systematic review, and the meta-analysis of the raw data. These data can then be linked to different pathway/protein interaction databases such as “Reactome” and “String” to further enhance our knowledge on the complex pathophysiology of IBD. This cycle is of course interactive and could lend into the possibility of discovering new cellular pathways as well as new functional roles for existing proteins, which can be further confirmed through targeted methodologies. An example of the value is the study of Kirov et al. who analysed the proteomics raw data deposited to the ProteomeXchange Consortium via the PRIDE ProteomXchange repository with the dataset identifier PXD001608 [186]. The findings of the(ir) initial publication (Bennike et al. [113]) were developed further by integrating published transcriptome data which indicated compensatory regulation at transcription levels of the ECM proteins. Key protein changes were confirmed by immunoblot analysis [186]. In terms of integrating omic data sets, there are two methods of data integration: horizontal and vertical meta-analyses which integrate multiple data sets of a similar type or of different types, e.g., transcriptomics and genomics [187]. Integrating datasets require careful assessment of compatibility (sample type, data format, and clinical metadata). For ohmic data sets, similar considerations apply with the added caveat that corresponding time points may not be appropriate as changes in mRNA are not necessarily concurrent or in the same direction of change to proteomic or metabolomics data.

It is increasingly recognised that proteoforms of individual proteins, arising from the combination of sequence variants and posttranslational modifications (including cleavage products), can be altered in level with respect to the specific disease state [172, 188]. This requires more exploration in IBD and the application of top-down methods as exemplified by analysis of specific proteoforms associated with Alzheimer's disease [189].

It is well recognised that proteome analysis, while of value, should be integrated with GWAS, epigenomic, transcriptomic, metabolomic, and microbiomic information to build a comprehensive molecular map of IBD. In terms of the microbiome, the emerging field of metaproteomics is enabling understanding of the host-microbiome in relation to IBD pathogenesis and microbial management-based therapeutic strategies. There are considerable technical challenges resulting in part from the complexity arising from studying, in effect, multiple species from the same sample: hundreds of bacterial species may be present in varying amounts within the clinical sample. Considerable progress has been made, as reviewed by Starr et al. [190]. A key development in proteomic tools includes the stable isotopically labelled microbiota (SILAMi) technique to study intestinal metaproteomes [191] and the MetaPro-IQ database for high-efficiency protein identifications of gut microbiome samples [192]. Development of tools to determine relationships between gut microbiomes, particularly transkingdom correlation analysis of mitochondrial protein expression, enabled the investigation of whether intestinal microbiomes associated with CD are causative or reflective of chronic long-term inflammation. The proteomic findings indicated mitochondrial dysfunction with downregulation of proteins implicated in H_2_S detoxification while the relative abundance of H_2_S microbial producers is increased. The bacterium, *A. parvulum*, which induces colitis *in vivo* in IBD IL-10-deficient mice (a mouse model of IBD) was demonstrated as a central hub of H2S production, with disease only occurring in the presence of other commensal microbiota [193]. Further metaproteomic analysis of mucosal luminal interface aspirates reveals that microbial proteins related to oxidative stress responses are upregulated, in intestinal extracellular vesicles of IBD cases, which correlate with abnormal host-microbiota interactions [194]. Metatranscriptomics can provide complementary insights into community interactions and disease-specific alterations.

In terms of biomarker panels, biomarkers can be molecules other than proteins; an example is mitochondrial (mt) DNA which is a potential mechanistic biomarker, with mtDNA-TLR9 proposed as a therapeutic target in IBD [195]. N-Formylated peptides were also identified by MS in this same study of intestinal mucosal mitochondrial damage and mtDNA release in active IBD. IgG Fc-glycosylation status has also been proposed for biomarker purposes [196].

In terms of clinical translation, state-of-the-art MS instrumentation is often more suited to research than clinical labs, but portable rapid nanosensing and monitoring platforms are coming online, as exemplified for detection and quantification of two tuberculosis-specific biomarkers (CFP-10 and ESAT-6) in blood samples [197]. Novel assays such as the proximity extension assay (PEA) enable rapid sample profiling which utilises the high sensitivity and specificity of polymerase chain reaction. This one-step, multiplex protein quantification method uses a pair of DNA oligonucleotides linked to antibodies directed to the target protein. The value of the approach has been demonstrated for detection of colorectal neoplasias [198] which has been applied to develop serum biomarker signatures for IBD, as part of the IBD Character Consortium (https://cordis.europa.eu/project/rcn/106191/reporting/en). In general, MS methods can complement traditional antibody-based methods and provide an alternative where antibodies are not available with potential for multiplex analysis for monitoring multiple proteins/sample that arise from discovery-based experiments: from proteomics, transcriptomics, and metaproteomics.

## Figures and Tables

**Table 1:** Quantitative proteomic workflows - examples of application, including the benefits and drawbacks of various gel and gel free proteomic methods.

Mode	Technique	Typical methods	Brief description	Quantification mode and benefits	Drawbacks	Example application to IBD research
Discovery	Two-dimensional electrophoresis [199]	2DE, DIGE	Gel-based separation of proteins employing immobilized pH gradients and polyacrylamide gels. Protein spot patterns compared between gels for each sample to identify differences in protein (spot) abundance between samples. DIGE technology uses spectrally resolvable fluorescent dyes to label up to 3 samples with different dyes and separate them on the same gel, for intra-gel quantitation.	Relative quantification, a low-cost approach to protein separation and sample analysis. Applicable to analysis of proteoforms [39]	Protein identification requires an additional MS step	*Samples*: serum profiles of the early-stage vs. acute-stage CD in comparison to healthy controls. *Key finding*: data suggest C3c proteolytic isoform expression in Crohn's disease could be disease-specific [188]

Discovery	Metabolic labelling	SILAC [200], Super-SILAC[201], and SILAMi [191]	*In vivo* protein labelling. Use of heavy, medium, and light isotopes allows discrimination from unlabeled (light) and thus relative quantification of peptides between samples	Relative quantification at MS level. Super-SILAC employs heavy reference standard enabling use of reference cell lines in patient studies	Not suitable for clinical material unless employing Super-SILAC for comparison to a common reference sample (cell line panel). Limited to comparison of 3 sample types (light, medium, and heavy SILAC labels)	*Samples*: paediatric control and biopsies of patients with CD and UC. Super-SILAC-based study. *Key finding*: identification of a panel of protein biomarkers to differentiate patients with CD from those with UC [177, 178]

Discovery	Chemical tagging [43]	iTRAQ, TMT [202–204]	*In vitro* labelling at the peptide level achieves simultaneous protein identification and quantification in multiplex format	Relative quantification at the MS2 level based on specific reporter ion intensities [203, 204]. Multiplex capability facilitates inclusion of replicates	Underestimation of fold change in relative protein amounts	*Sample type*: biopsy samples from UC patient groups differing in cancer risk and inflammatory status and healthy control. *Key finding*: acute inflammation resulted in reduced levels of intermediate filament proteins (keratins 8,18, and 19 and vimentin) compared to control and noninflamed mucosa as determined using iTRAQ analysis [172]

Discovery and targeted	Label-free methods (LFQ) [205, 206]	Used in Data Dependent acquisition and data-independent acquisition (MS^E^ [207, 208], SWATH) [209, 210] approaches	Peptide intensity-based measurements, based on peak integration or spectral counting at MS level.	Relative quantification across multiple samples	Quantification achieved from independent sample runs. It is thus dependent on highly reproducible HPLC separations prior to MS. MSE and SWATH experiments enable retrospective data mining for specific peptides for relative quantification.	*Sample type:* colon tissue biopsies from normal, active, and inactive UC, inflammatory polyps, active and inactive CD. *Key finding*: three novel proteins, PRG2, LCP1, and PSME1, identified as candidate biomarkers signifying active CD [104]

Targeted	Targeted proteomic approaches [211]	Selected reaction monitoring (SRM) and related parallel reaction monitoring approach. When SRM is applied to investigate multiple product ions, this method is referred to as MRM	Quantification is based on detection and measurement of quantotypic peptides that represent the protein, based on a unique (proteotypic) amino acid sequence that is quantotypic, i.e., unique to the protein and stoichiometric to the amount of corresponding protein.	Relative quantification absolute quantification by reference to synthetic light and 13C/N15-labelled heavy peptides. Assays can be in multiplex format.	Cost of synthetic peptides and method optimisation	*Sample type*: plasma–low mass peptides. *Key finding*: peptides derived from secreted phosphoprotein 24 (SPP24) differentiates IBD from control. Active and quiescent disease can differentiate in UC and CD by secretogranin 1 and alpha-1-microglobulin [213]

2DE: two-dimensional gel electrophoresis; DIGE: difference gel electrophoresis; SILAC: stable isotope labeling in cell culture; SILAMi: stable isotopically labelled microbiota; TMT: tandem mass tag; iTRAQ: isobaric tags for relative and absolute quantitation; MSE: method of MS analysis that records exact mass precursor and fragment ion information while simultaneously obtaining accurate quantitation for each component peptide; SWATH: sequential window acquisition of all theoretical spectra; LFQ: label-free quantification; SRM: selected reaction monitoring; MRM: multiple reaction monitoring; PRM: parallel reaction monitoring.

**Table 2:** High-throughput proteomic studies in IBD.

Proteomic studies in IBD	References	Analytical technique(s)	Type of study (cell line, animal, human)	Significant finding(s)
Biofluid proteomics	Meuwis et al. 2007 [50]	SELDI-TOF-MS	Human	Identification of PF4, MRP8, FIBA, and Hpa2 as potential biomarkers of disease activity in IBD
	Meuwis et al. 2008 [61]	SELDI-TOF-MS	Human	Positive correlation between PF4 level and nonresponse to infliximab therapy in CD
	Kanmura et al. 2009 [62]	Protein array technology coupling ProteinChip system with SELDI-TOF/MS	Human	Identification of 3 increased in abundance proteins (HNP 1–3) in the serum of active UC patients as new biomarkers of UC disease activity
	Hatsugai et al. 2010 [71]	2DE, discriminant analysis (OPLS-DA), and MALDI-TOF MS/MS	Human	Identification of a panel of proteins that could discriminate between UC and CD including ANXA6, PRDX2, ABHD14B, cyclophilin A, CA2, ACTB, and S100A9
	Gazouli et al. 2013 [72]	2DE and MALDI-TOF-MS	Human	Identification of a panel of increased in abundance proteins in the primary infliximab nonresponsive patients including: APOA1, APOE, CO4B, PLMN, TRFE, APOH, and CLUS in comparison to infliximab-induced remission patients
	Vaiopoulou et al. 2015 [63]	2DE and MALDI-TOF-MS	Human	Overexpression of ceruloplasmin and apolipoprotein B-100 (APOB) in children and clusterin in adults with CD
	Viennois et al. 2015 [76]	2DE and MALDI-TOF/TOF MS	Murine	Identification of altered expression of proteins specific to intestinal inflammation development (PZP, COL1A1, and PRDX2), IL10^−/−^ murine model inflammation (PZP, PRDX2), arthritic inflammation development (serum amyloid P-component and transthyretin), and nonspecific inflammation (HP, ITIH4, HPX, C3, and A1BG)

Tissue proteomics	Barcelo-Batllori et al. 2002 [131]	2DE and MALDI-TOF-MS	Cell line and human	Increase of indoleamine-2,3-dioxygenase in the purified IEC from IBD patients relative to normal controls
	Hsieh et al. 2006 [99]	2DE and MALDI-TOF-MS	Human	Identification of 19 altered proteins expression (13 decreased in abundance and 6 increased in abundance) in the active compartment of UC mucosa relative to control. Altered regulation of mitochondrial proteins raising the possibility of mitochondrial dysfunction in UC
	Liu et al. 2007 [137]	2DE, MALDI-TOF-MS, and real-time RT-PCR	Murine	Alteration of a series of apoptosis-related proteins (increased in abundance IL-12 p40 and decreased in abundance CARD, PYD domain-containing protein, and proteasome activator complex subunit 2), proteins involved in cell growth, differentiation, and signal transduction (increased in abundance NDPKs and E2N), infl ammatory factors (increased in abundance MRP14), and metabolism/oxidative stress response-related proteins (increased in abundance ATP-citrate synthase, phosphoglycerate mutase, and dismutase) in lymphocytes from a rat colitis model
	Shkoda et al. 2007 [81]	2DE and MALDI-TOF MS	Human	Identification of several differentially expressed proteins in the IEC from active IBD patients relative to controls (including L-lactate dehydrogenase, NADPH prostaglandin E2 reductase, keratin 19, and Rho GDI *α*)
	Fogt et al. 2008 [92]	2DE and LC-MS/MS	Human	Identification of increased expression of proteins that are mainly involved in inflammation and repair (proto-cadherins, *α*-1 antitrypsin, tetratricopeptide repeat domains, caldesmon, and mutant desmin) in the active UC tissue relative to the inactive proximal mucosa
	Brentnall et al. 2009 [121]	HPLC, Q-TOF MS/MS, and iTRAQ labelling	Human	Identification of differential protein expression relating to mitochondria (CPS1 and S100P) associated with UC neoplastic progression
	Araki et al. 2009 [129]	2DE and LC-MS/MS	Cell line and human	Identification of overexpression of HSP47 in UC-associated cancer relative to sporadic colon cancer tissues
	May et al. 2011 [126]	Label-free LTQ-Orbitrap hybrid MS coupled with HPLC	Human	Identification of two differentially expressed mitochondrial proteins (TRAP1, CPS1) associated with UC neoplastic progression, which was found in both dysplastic and nondysplastic tissues
	Zhao et al. 2011 [117]	2DE and MALDI TOF/TOF MS	Human	Detecting P-p38 increase and MAWBP/galectin-3 decrease in active UC compared to normal controls; proposing P38 MAPK pathway involvement in UC disease activity
	M'Koma et al. 2011 [78]	Histology-directed MALDI-MS(tissue imaging)	Human	Discriminatory colonic submucosa proteomic profile between UC and Crohn's colitis via investigation of MS spectral peaks
	Poulsen et al. 2012 [102]	2DE and MALDI-TOF MS together with PCA analysis	Human	Identification of 33 individual proteins with altered expression in the active UC mucosa relative to proximal inactive mucosa including proteins involved in energy metabolism (triosephosphate isomerase, glycerol-3-phosphate-dehydrogenase, alpha enolase, and L-lactate dehydrogenase B-chain) and in oxidative stress (superoxide dismutase, thioredoxins, and selenium-binding protein)
	Kwon et al. 2012 [115]	2DE and MALDI-TOF/TOF MS	Human	Identification of 3 proteins with altered expression in UC and tuberculous colitis (TC) (mutant *β*-actin, *α*-enolase, Charcot-Leyden crystal) relative to control, suggesting *α*-enolase as a candidate biomarker for differential diagnosis of TC and UC
	Barnett et al. 2013 [149]	2DE and LC-MS	Murine	Reduced abundance of proteins associated with immune and inflammatory response as well as fibrinogenesis pathways, and increased abundance of those associated with xenobiotic metabolism pathways in response to GrTP
	Seeley et al. 2013 [80]	Histology-directed MALDI-MS(tissue imaging)	Human	Identification of a support vector machine algorithm consisting of 25 peaks able to differentiate spectra from CC and UC with 76.9% spectral accuracy
	Han et al. 2013 [104]	Label-free LC-MS	Human	Suggestion of 3 proteins as potential biomarkers for active CD, including PRG2, LCP1, and PSME1
	Zhou et al. 2013 [128]	2DE, WB, and MALDI MS	Human	Identified 6 differentially expressed proteins (prohibitin, calreticulin, apolipoprotein A-I, intelectin-1, protein disulphide isomerase, and glutathione s-transferase Pi) in active CD mucosa relative to the adjacent normal-looking mucosa

	Bennike et al. 2015 [113]	Label-free (LFQ) proteomic analysis	Human	Comparing UC to normal biopsies identified proteins with statistically significant altered abundance in the UC biopsies to be from neutrophils and associated with the formation of neutrophil extracellular traps (NETs) suggesting stimulation of innate immunity.

Organelle proteomics	O'Morain et al. 1985 [161]	2DE and MS	Human	Identification of specific alterations in the principal subcellular organelles, especially the plasma membrane, lysosomes, and mitochondria in IBD in comparison to control
	Nanni et al. 2009 [73]	1D SDS-PAGE and nano-LC ESI/Q-TOF-MS with targeted MS/MS analysis	Human	Identification of certain decreased in abundance (Annexin A1, malate dehydrogenase) and increased (different histones and ubiquitin in the nuclear fraction; tryptase alpha-1 precursor in the cytosolic fraction; ATP synthase subunit beta and heat shock 70 kDa protein-5 in the fraction containing membranes) proteins in CD patients relative to controls
	Nanni et al. 2009 [212]	LC ESI/Q-TOF-MS with targeted MS/MS analysis	Human	Suggestion of a specific serum exoprotease activity with proinflammatory properties in CD
	Corfe et al. 2015 [172]	HPLC, ESI/Q-TOF MS-MS, and iTRAQ labelling	Human	Reduced levels of K8, K18, and K19 and vimentin (VIM) in acute distal UC mucosa compared to controls and noninflamed proximal mucosa; reduced levels of IF–associated proteins in UC with PSC and UC with dysplasia; increased levels of K8, K18, and K19 and VIM in longstanding pancolitis in remission relative to controls and recent-onset colitis in remission

	Denadai-Souza et al. 2018 [167]	MS-based functional proteomic analysis employing activity-based probes	Human	Profiling and identification of active serine proteases were performed to monitor availability of enzyme active sites in colonic tissues from CD or UC patients. Seven serine proteases including cathepsin G, plasma kallikrein, plasmin, tryptase, chymotrypsin-like elastase 3A, thrombin, and aminopeptidase B were identified, of which cathepsin G and thrombin were overactive in IBD patient samples compared to healthy controls.
